# Impact of Different Personal Protective Clothing on Wildland Firefighters' Physiological Strain

**DOI:** 10.3389/fphys.2017.00618

**Published:** 2017-08-24

**Authors:** Belén Carballo-Leyenda, José G. Villa, Jorge López-Satué, Jose A. Rodríguez-Marroyo

**Affiliations:** ^1^Department of Physical Education and Sports, Institute of Biomedicine, University of León León, Spain; ^2^Empresa de Transformación Agraria Madrid, Spain

**Keywords:** heat stress, thermal strain, thermophysiological response, core temperature, protective clothing

## Abstract

Wildfire firefighting is an extremely demanding occupation performed under hot environment. The use of personal protective clothing (PPC) is needed to protect subjects from the thermal exposure. However, the additional use of PPC may increase the wildland firefighters' physiological strain, and consequently limit their performance. The aim of this study was to analyze the effect of four different PPC on the physiological strain of wildland firefighters under moderate conditions (30°C and 30% RH). Eight active and healthy wildland firefighters performed a submaximal walking test wearing a traditional short sports gear and 4 different PPC. The materials combination (viscose, Nomex, Kevlar, P-140 and fire resistant cotton) used during the PPC manufacturing process was different. During all tests, to simulate a real scenario subjects wore a backpack pump (20 kg). Heart rate, respiratory gas exchange, gastrointestinal temperature, blood lactate concentration, perceived exertion and temperature and humidity underneath the PPC were recorded throughout tests. Additionally, parameters of heat balance were estimated. Wearing a PPC did not cause a significant increase in the subjects' physiological response. The gastrointestinal temperature increment, the relative humidity of the microclimate underneath the PPC, the sweat residue in PPC, the sweat efficiency, the dry heat exchange and the total clothing insulation were significantly affected according to the PPC fabric composition. These results suggest that the PPC composition affect the moisture management. This might be taken into account to increase the wildland firefighters' protection in real situations, when they have to work close to the flames.

## Introduction

Wildfire firefighting is an extremely demanding occupation (Ruby et al., 2002; Cuddy et al., 2015) that mainly takes place during the summer season. Activities performed during wildfire suppression require work with hand tools of different weight (3–20 kg) (Rodríguez-Marroyo et al., 2012) and they are usually performed under difficult conditions, such as inhaling smoke (Wegesser et al., 2009), working on steep terrain (Brotherhood et al., 1997) and in hot environments (Raimundo and Figueiredo, 2009; Rodríguez-Marroyo et al., 2012). Collectively, all these circumstances contribute to the high physiological strain observed during wildfires suppression (Rodríguez-Marroyo et al., 2012).

Performing demanding tasks in hot environments is associated with an increased heat stress (Gonzalez-Alonso et al., 1999; Cheuvront et al., 2010). The additional use of personal protective clothing (PPC) may increase the wildland firefighters' thermal strain (Bruce-Low et al., 2007), and consequently limit their performance (Selkirk and McLellan, 2004; Taylor et al., 2012). PPC affects the heat-dissipating thermoregulatory mechanisms since they limit the heat loss and vapor transfer between the skin and the environment (Holmér, 2006). Nonetheless, PPC protects subjects from a wide variety of work-related hazards mainly from the thermal exposure (Nayak et al., 2014). They are manufactured according to security standards (ISO, 2003), where technical requirements of the fabrics are specified. However, this certification does not take into account the process of making the suits, which does not allow to directly extrapolate the response that these fabrics will induce in thermoregulation once they constitute a complete suit and this is carried by a person. The design and materials combination during the PPC manufacturing process may affect their performance on the thermoregulatory response (Havenith and Heus, 2004). The degree of PPC's thermal and vapor insulation will depend on the clothing thickness, trapped air layers and fiber characteristics (e.g., weave, coatings and membranes) (Havenith, 2002).

It seems clear that PPCs have to provide a specific protection and minimize subjects' thermal and physiological strain in order to avoid injuries and not limit their performance. There is considerable research regarding the effect of different PPC on the thermoregulatory response of structural firefighters (Sköldström, 1987; Smith and Petruzzello, 1998; Selkirk and McLellan, 2004; Holmér et al., 2006; Bruce-Low et al., 2007), chemical (Wen et al., 2015), military (Montain et al., 1994) and industry (Poirier et al., 2015) situations. This contrasts with the paucity of studies carried out with wildland firefighters. To the best of our knowledge, an earlier study had analyzed wildland firefighters' response to different PPC (Budd et al., 1997). Budd et al. (1997) compared two different PPC, one thicker and more encapsulated vs. a lighter and more open one. However, these authors did not assess the physiological impact of the PPC on wildland firefighters vs. a control experimental condition (e.g., sports gear). Therefore, the aim of this study was to analyze the effect of four different PPC, according to their fabric composition, on the physiological strain of wildland firefighters during moderate exercise intensity (i.e., 250 W·m^−2^) and under warm environmental conditions (i.e., 30°C, 30% RH).

## Methods

### Participants

Eight active and healthy male wildland firefighters (mean ± SD; age 30.8 ± 8.4 years, height 1.79 ± 0.06 m, body mass 76.9 ± 10.8 kg, maximal oxygen uptake 55.4 ± 9.1 ml·kg^−1^·min^−1^, and body surface area 1.9 ± 0.2 m^2^) were involved in this study. Subjects performed endurance exercise (45–60 min/training session) three times per week as part of their scheduled training. Written informed consent was obtained from the volunteers before starting the study. The study protocol was developed in accordance with the guidelines of the Helsinki Conference for research on human subjects and was approved by the Ethics Committee of the University of León, Spain.

### Experimental design

Each subject performed six trials over six separate testing sessions. Trials were separated by at least 48 h, during which participants were asked to refrain from strenuous exercise. The first trial was a maximal incremental test to determine subjects' characteristics and their exercise capacity (Bruce, 1971). During the second to sixth trial, subjects performed, in a balanced Latin Square design, a 120 min submaximal test wearing a traditional short sports gear (i.e., shorts, and cotton t-shirt, underwear and socks) and 4 different PPC. All PPC adhered to (ISO, 2003) and are currently used by Spanish wildland firefighters. Although wildland firefighters' personal protective equipment includes items such as helmet, gloves, goggles and mid-calf leather boots, they were not used during the trials. The same clothing (i.e., cotton t-shirt, underwear and socks) was worn under PPC during all trials. Garments' specifications are showed in Table 1. During all tests, to simulate a real scenario subjects wore a backpack pump (20 kg), which is routinely used during wildfire firefighting (Rodríguez-Marroyo et al., 2012). In addition, the same running shoes (250–300 g per shoe) were used in every testing session.

**Table 1:** Personal protective clothing (PPC) characteristics.

**Layer**		**PPC#1**	**PPC#2**	**PPC#3**	**PPC#4**
		**Single**	**Reflective double layer on shoulders**	**Single**	**Single**
Composition	FR viscose (%)	65	65	56	
	Nomex (%)	30	30	34	
	Kevlar (%)	5	5	8	
	P-140 (%)			2	
	FR cotton (%)				100
Mass (g)		1,460	1,560	1,440	1,000
Surface mass (g·m^−2^)		270	270	225	310
Total mass (kg)^*^		79.6 ± 7.4	79.5 ± 6.6	79.7 ± 7.0	78.1 ± 6.5
Fabric thermal resistance (m^2^·K·W^−1^)		0.0192	0.0192	0.0213	0.0260
Fabric evaporative resistance (m^2^·Pa W^−1^)		3.79	3.79	3.45	3.51

*FR, fire resistant*.

* *Subjects mass while wearing the PPC*.

### Tests protocol

All tests were performed on a treadmill (h/p/cosmos pulsar, h/p/cosmos sports and medical GMBH, Nussdorf-Traunstein, Germany). Each test was preceded by a 10 min warm-up at 60% of maximum heart rate (8–10 km·h^−1^) and 5 min of stretching. The maximal test was performed according to the protocol described by Bruce (1971). The test started with a speed of 2.5 km·h^−1^ and a slope of 10%. The speed and grade were incremented every 3 min until volitional exhaustion.

The submaximal tests were performed at the same time of the day (between 12:00 and 16:00 h) in a laboratory under climate-controlled conditions. Air temperature and relative humidity were maintained at 30°C and 30%, respectively, simulating those analyzed in real wildland fires (Rodríguez-Marroyo et al., 2012). The protocol consisted of 6 sets of walking at 6 km·h^−1^ and a slope of 1% with 5 min passive rest periods in between. Each set duration was 15 min, except for the first set that was 20 min, so the total test length was 120 min. During recovery periods, 0.15 ml·kg^−1^ of water every 1 min of exercise at 15°C (Selkirk and McLellan, 2004) was administered to prevent that subjects' dehydration limited the sweat rate (Cheuvront et al., 2010). The protocol used in this study was based on previous studies (Selkirk and McLellan, 2004) and the selected speed allowed subjects to perform an exercise intensity between 60 and 70% of the maximal heart rate, which simulates wildland firefighters' working conditions (Rodríguez-Marroyo et al., 2012).

### Measurements

ECG monitoring (Medisoft Ergocard, Medisoft Group, Sorinnes, Belgium) was performed throughout the maximal test to detect heart problems. During both maximal and submaximal tests, the heart rate (HR) response and the respiratory gas exchange was continuously measured every 5-s (RS800, Polar Electro Oy, Kempele, Finlandia) and breath-by-breath (Medisoft Ergocard, Medisoft Group, Sorinnes, Belgium), respectively. VO_2max_ was accepted as the highest 30-s moving average.

Gastrointestinal temperature was recorded throughout submaximal trials using a Jonah intestinal temperature capsule (VitalSense, Phillips Respironics, Bend, OR, USA) which was ingested at least 8 h before the beginning of trials (Wen et al., 2015). Temperature and humidity underneath the PPC were also measured (Termoregister TR-72U, T and D, Japan). Data logger was placed at the sternum level, between the cotton t-shirt and PPC. Temperature and humidity data, as well as HR and VO_2_ data from the last 5 min of each submaximal exercise stage, were considered representative measurements of each stage. The gastrointestinal temperature and HR data were used to calculate the physiological strain index (PSI) throughout the trials according to Tikuisis et al. (2002).

Capillary blood samples were taken from a previously hyperemized earlobe to measure blood lactate concentration (Lactate Scout, Senslab, Leipzig, Germany) after the end of each submaximal stage. During the last 30-s of each exercise stage, the Rating of Perceived Exertion (RPE) was recorded using the Borg scale (6–20) (Borg, 1982). The scale was explained and administered by the same researcher, asking about subjects' perceived exertion using the same question. A cue card was located in front of subjects to allow immediate reference to the scale. Additionally, subjects' moisture sensation was recorded at the end of each trial, using a categorical scale (1–9) (Havenith and Heus, 2004). Verbal anchors associated with 1 and 9 were identified with *slightly moist* and *totally soaked*, respectively.

Subjects, in underwear, and each clothing component were separately weighted (50K150, COBOS, Hospitalet de Llobregat, Barcelona, Spain) at the beginning and the end of each submaximal trial. This allows calculating the total sweat production, sweat residue and sweat evaporation (Havenith and Heus, 2004; Kofler et al., 2015). Total sweat was corrected for the fluid intake. Water loss through breathing was neglected and was assumed to be similar between trials (Saunders et al., 2005). Finally, the sweat efficiency was calculated as the ratio between sweat evaporation and total sweat (Havenith and Heus, 2004).

Heat balance of the body was estimated using a method of partitional calorimetry summarized in equation 1 (Bröde et al., 2008):

(1) $$\text{S}=\text{M}-\text{W}\pm \text{DRY}-{\text{E}}_{\text{sk}}-\text{RES}$$

Components of the equation were heat storage (S), metabolic energy production (M), effective mechanical work (W), heat loss through evaporative and convective heat exchange via respiration (RES = E_res_ + C_res_), evaporative heat loss (E_sk_), and dry heat loss (DRY = C + R + K). All heat balance parameters were calculated in W·m^−2^. The components were estimated and served only to substantiate the results.

The rate of metabolic heat production was calculated from measured respiratory quotient (RQ) and VO_2_ (L·min^−1^) and the body surface area (A_D_; m^2^) calculated using DuBois formula (DuBois and DuBois, 1916), as shown below in Equation (2) (Gagge and Gonzalez, 1996):

(2) $$\text{M}=[0.23(\text{RQ})+0.77]\times 5.873\times {\text{VO}}_{2}\times (60/{\text{A}}_{\text{D}})$$

Effective mechanical work was calculated using acceleration due to gravity (9.8 m·s^−2^), the dressed mass of participants (m_d_; kg), the speed (*v*; m·s^−1^) and the grade fraction (F) of the treadmill and A_D_, using Equation (3) (McLellan et al., 1996):

(3) $$\text{W}=9.8\times {\text{m}}_{\text{d}}\times \text{v}\times \text{F}\times {{\text{A}}_{\text{D}}}^{-1}$$

The respiratory heat loss components C_res_ and E_res_ were calculated using Equations (4) and (5), respectively (Bröde et al., 2008):

(4) $${\text{C}}_{\text{res}}=1.516\times 10^{-3}\times \text{M}\times (28.56-0.641\times {\text{P}}_{\text{a}}-0.885\times {\text{T}}_{\text{a}}$$

(5) $${\text{E}}_{\text{res}}=1.27\times 10^{-3}\times \text{M}\times (59.34-11.63\times {\text{P}}_{\text{a}}+0.53\times {\text{T}}_{\text{a}}$$

where P_a_ is the atmospheric pressure in Pascals, T_a_ is the ambient temperature in °C and M is the rate of metabolic heat production in W·m^−2^, calculated with Equation (3).

S was calculated as (ΔT_b_ × Δt^−1^) × BM × ${\text{A}}_{\text{D}}^{-1}$ × c_p_. ΔT_b_ × Δt^−1^ in °C·s^−1^ was described as the rate of change of body temperature accounting for tests duration (s), c_p_ represented the specific heat of body tissue (3,480 J) and BM, body mass in kg. As skin temperature (T_skin_) was not measured, the chest temperature (T_chest_) of the microclimate underneath PPC served as an indirect marker of T_skin_ (Kofler et al., 2015). Mean body temperature (T_b_) in °C was estimated by 4:1 ratio of gastrointestinal temperature (T_GI_) and T_chest_ as T_b_ = 0.8 × T_GI_ + 0.2 × T_chest_, recommended for warm environments (Bröde et al., 2008).

E_sk_ corrected for respiratory loss was estimated as $\lambda \times \left({\text{m}}_{\text{e}}\times \Delta {\text{t}}^{-1}\right)\times {\text{A}}_{\text{D}}^{-1}-{\text{E}}_{\text{res}}$. Where m_e_ is the evaporative sweat loss (g) with Δt denoting measurement time (s), λ the enthalpy of evaporation (2,430 J·g^−1^) and E_res_ is the respiratory heat evaporation calculated using Equation (5). DRY resulted by solving the heat balance equation with the other known components using Equation (6) (Bröde et al., 2008):

(6) $$\text{DRY}=\text{ M}-W-{\text{E}}_{\text{sk}}-\text{RES}-\text{S}$$

Additionally, total insulation (I_t_) of clothing was estimated through the equation (T_chest_ - T_a_) × DRY^−1^. This estimation was also less precise, like the calculation of S because of using T_chest_ instead of T_skin_ (Kofler et al., 2015).

### Statistical analysis

The results are expressed as mean ± standard deviation (*SD*). The assumption of normality was verified using the Shapiro-Wilk's test. The variables analyzed throughout the submaximal trials (VO_2_, ventilation, HR, blood lactate concentration, gastrointestinal temperature, PSI, micro environment temperature, relative humidity, and RPE) were compared using a repeated two-way ANOVA with two within-subject factors (clothing and time). A one-way ANOVA with repeated measures was applied to calculate differences between trials when subjects' gastrointestinal temperature increment, moisture sensation, and the different parameters of heat balance and sweat were studied. When a significant *F*-value was found, Bonferroni's test was used to establish significant differences between means. The assumption of sphericity was checked using the Mauchly's test when this assumption was violated the Greenhouse-Geisser adjustment was performed. Values of *p* < 0.05 were considered statistically significant. SPSS V.19.0 statistical software (SPSS Inc., Chicago, Illinois, USA) was used.

## Results

**Figure 1:**
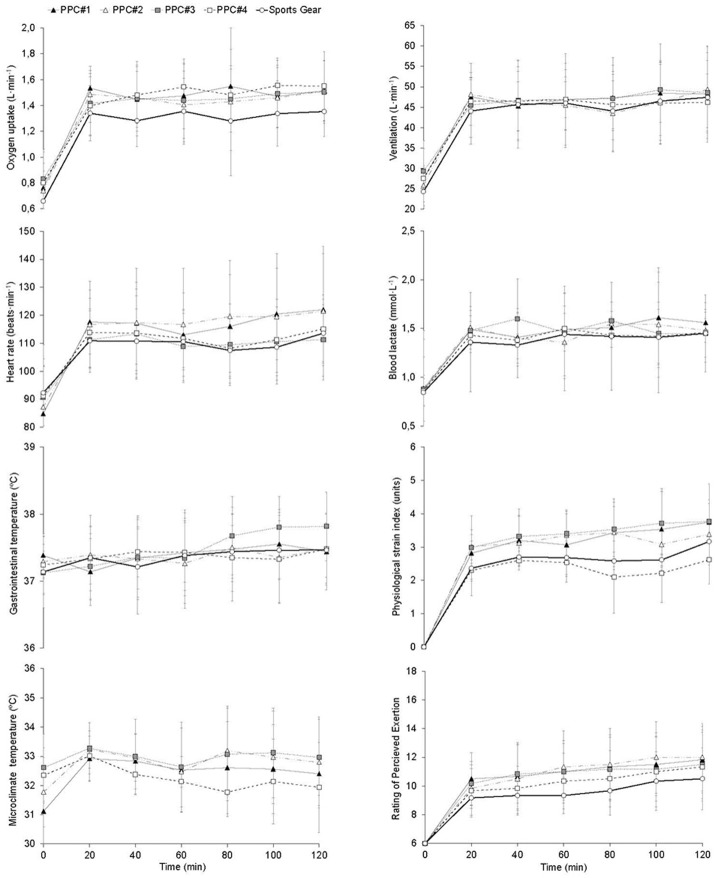
Comparative responses of oxygen uptake, ventilation, heart rate, blood lactate concentration, gastrointestinal temperature, physiological strain index, temperature of the microclimate underneath the personal protective clothing (PPC), and rating of perceived exertion during the different trials.

The interaction between clothing condition and time was not significant for VO_2_, ventilation, HR, blood lactate, gastrointestinal temperature, PSI, temperature underneath the PPC, and RPE (Figure 1). The mean values analyzed during the trials were: 1.5 ± 0.3 L·min^−1^, 48.4 ± 8.5 L·min^−1^, 114 ± 15 bpm, 1.5 ± 0.2 mmol·L^−1^, 37.4 ± 0.5°C, 3.2 ± 0.7 units, 32.7 ± 1.2°C and 10.7 ± 2.0, respectively. Likewise during the trials no significant main effect of time condition was present on these variables (Figure 1). However, the gastrointestinal temperature increment was significantly higher with PPC#3 (0.7 ±0.3°C) than those analyzed with the others PPC (0.2 ± 0.3, 0.2 ± 0.5 and 0.2 ± 0.3°C for PPC#1, PPC#2, PPC#4, respectively) and sports gear (0.3 ± 0.3°C).

**Figure 2:**
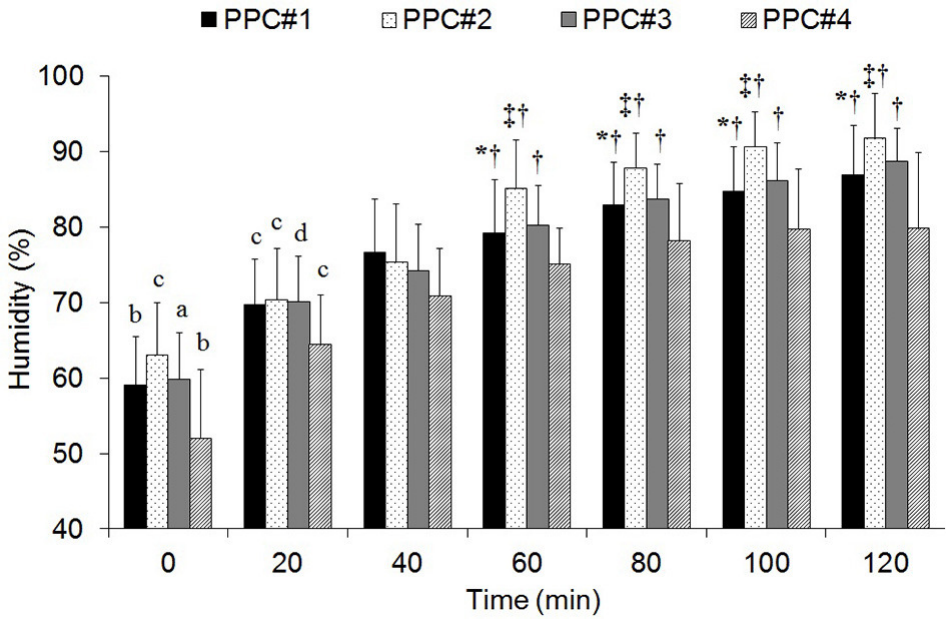
Relative humidity pattern recorded during the trials performed with the different personal protective clothing (PPC). ^*^Differences with PPC#2 (*p* < 0.05). ^‡^Differences with PPC#3 (*p* < 0.05). ^†^Differences with PPC#4 (*p* < 0.05). ^a^Differences with 20–120 min (*p* < 0.05). ^b^Differences with 40–120 min (*p* < 0.05). ^c^Differences with 60–120 min (*p* < 0.05). ^d^Differences with 100–120 min (*p* < 0.05).

The relative humidity of the microclimate underneath the PPC was higher (*p* < 0.05) in PPC#2, PPC#3 than PPC#4 (81.2 ± 4.7 and 77.7 ± 5.0 vs. 71.7 ± 4.9%). From the 60th minute of trail, the lowest (*p* < 0.05) values were analyzed in PPC#4 (Figure 2). The humidity data increased more rapidly during the first part of the trials (i.e., 0–60 min), and it slowed during the second hour (i.e., 60–120 min) (Figure 2). Collectively, significant differences (*p* < 0.05) between the data analyzed at 5–20 min (~63% RH) vs. 60–120 min (~84% RH) were found.

The total sweat production was similar in all PCC and when subject wore the sports gear (Table 2). The sweat residue in underwear was similar in all tests. However, the sweat residue in PPC#1 was the lowest (*p* < 0.05), and as a consequence the sweat efficiency of PPC#1 was the highest (*p* < 0.05) of all PCC analyzed in this study. Indeed, the lower (*p* < 0.05) subjects' moisture sensation was analyzed with the PPC#1 (5.6 ± 0.3, 7.6 ± 0.9, 7.0 ± 0.6, and 8.2 ± 0.5 for PPC#1, PPC#2, PPC#3, and PPC#4, respectively). The lowest value was obtained when subjects wore the sports gear (3.8 ± 1.0).

**Table 2:** Sweat measurements analyzed in this study (mean ± *SD*).

	**PPC#1**	**PPC#2**	**PPC#3**	**PPC#4**	**Sports gear**
Total sweat production (g)	1, 910 ± 360	2, 342 ± 450	2, 110 ± 390	1, 968 ± 370	1, 925 ± 447
Sweat residue in underwear (g)	367 ± 53	368 ± 70	361 ± 54	409 ± 61	335 ± 156
Sweat residue in garment (g)	178 ± 51^*^^‡^^†^	579 ± 278	545 ± 156	418 ± 124	
Sweat evaporation (g)	1, 514 ± 327	1, 395 ± 161	1, 274 ± 268	1, 189 ± 338^$^	1, 609 ± 251
Sweat efficiency (%)	74 ± 5^*^^‡^^†^^$^	61 ± 7^$^	59 ± 5^$^	58 ± 9	84 ± 8

*PPC, personal protective clothing*.

* *Differences with PPC#2 (p < 0.05)*.

‡ *Differences with PPC#3 (p < 0.05)*.

† *Differences with PPC#4 (p < 0.05)*.

$ *Differences with Sports Gear (p < 0.05)*.

Estimated parameters of heat balance are showed in Table 3. The dry heat exchange of PPC#2 and PPC#3 was significantly (*p* < 0.05) different from PPC#4. Total clothing insulation was significantly (*p* < 0.05) lower in PPC#1.

**Table 3:** Estimated parameters of heat balance analysis (mean ± *SD*).

	**PPC#1**	**PPC#2**	**PPC#3**	**PPC#4**
Heat storage (W·m^−2^)	4.5 ± 7.5	5.5 ± 14.0	8.1 ± 6.3	3.8 ± 6.0
Metabolic heat production (W·m^−2^)	250.5 ± 23.7	238.4 ± 26.1	243.2 ± 34.9	248.1 ± 22.4
Respiratory heat exchange (W·m^−2^)	10.2 ± 1.0	9.7 ± 1.0	9.9 ± 0.9	10.1 ± 0.9
Evaporative heat loss from skin (W·m^−2^)	251.5 ± 48.1	232.9 ± 30.5	210.9 ± 42.9	197.4 ± 57.7
Dry heat exchange (W·m^−2^)	15.8 ± 15.7	9.9 ± 12.4^†^	11.4 ± 6.7^†^	30.4 ± 24.1
Total clothing insulation (m^2^·°C·W^−1^)	0.08 ± 0.06^*^^‡^^†^	0.35 ± 0.38	0.31 ± 0.22	0.26 ± 0.36

*PPC, personal protective clothing*.

* *Differences with PPC#2 (p < 0.05)*.

‡ *Differences with PPC#3 (p < 0.05)*.

† *Differences with PPC#4 (p < 0.05)*.

## Discussion

The aim of this study was to investigate the effect of different PPC on the physiological strain of wildland firefighters. Contrary to our expectations, no significant differences in the cardiorespiratory variables analyzed between the ensembles were found (Figure 1). Previous studies (Baker et al., 2000; Dorman and Havenith, 2009; Wen et al., 2015) reported an increase of 10–20% in the physiological response (e.g., VO_2_) with the use of different PPC. Other studies (Sköldström, 1987; Taylor et al., 2012; Lee et al., 2014) analyzed a greater increase (>20%) in structure firefighters when they used the self-contained breathing apparatus, due to the rise in weight (10–20 kg) of the ensemble (Lee et al., 2014). The mean increase observed in this study when wearing the PPC was lower, ~12%. Possibly, this was consequence of the lower thermal insulation of the PPC used by wildland firefighters (~0.23 m^2^·K·W^−1^) (Raimundo and Figueiredo, 2009) vs. those of structure firefighters (0.47 m^2^·K·W^−1^) (Holmér et al., 2006), which have allowed for a greater heat dissipation. Additionally, in this study subjects performed the trials without gloves, helmet and boots. This conditioned their thermoregulatory response by increasing the body surface exposed to the environment and thus facilitate the heat loss (Holmér, 2006; Lee et al., 2014). Lee et al. (2014) did not find significant differences in the VO_2_ and HR of structure firefighters when they wore the protective equipment without gloves, helmet and boots vs. a sports gear.

The limited weight difference between the ensembles studied might have influenced in the physiological variables pattern. It has been reported that more than half of the increase in the metabolic rate is due to the PPC weight (Dorman and Havenith, 2009). Indeed, no differences in the cardiorespiratory response or RPE have been found when wearing a light-weight workwear compared to a control condition (Kofler et al., 2015). On the other hand, the subjects' aerobic fitness might attenuate the HR, VO_2_, gastrointestinal temperature and RPE response in the trials where the PPC were worn (Selkirk and McLellan, 2001). The VO_2max_ analyzed in this study was ~28% higher than that previously described (~43 ml·kg^−1^·min^−1^) in wildland firefighters (Phillips et al., 2011). Subjects with greater fitness present higher tolerance to effort under conditions of thermal stress wearing a PPC (Selkirk and McLellan, 2001).

Wearing a PPC in hot environments has been associated with an increased body temperature (Smith and Petruzzello, 1998; Bruce-Low et al., 2007). However, in moderate environmental conditions, such as those maintained in this study, no significant differences in gastrointestinal temperature were observed (Figure 1), despite the greater temperature elevation analyzed throughout the trial in PPC#3. Collectively, this findings are in agreement with those obtained in previous research performed under similar conditions with light-weight PPC (Wang et al., 2013; Kofler et al., 2015). Subjects' gastrointestinal temperature was below 38°C during all trials. This value has been informed as a limiting factor of performance during exercise in heat (Gonzalez-Alonso et al., 1999). Possibly, the temperature remained stable during the trials performed with PPC by the increase of the cardiovascular stress. In fact, the mean HR was ~5% higher (*p* > 0.05) when subjects wore the PPC. It may be speculated that this was due to the increase of the cutaneous circulation in order to favor the heat dissipation (Cheuvront et al., 2010). The HR pattern might also be reflecting the greater metabolic rate related to the weight difference between PPC and the sports gear configuration (Dorman and Havenith, 2009). The VO_2_ was higher (*p* > 0.05) by ~200 ml·min^−1^ with vs. without PPC (Figure 1).

Globally, we analyzed a difference of ~2.5°C between the temperature of the microclimate underneath the PPC and the outside temperature. However, the mean temperature of the microclimate of PPC#2 and PPC#3 was 0.8°C higher (*p* > 0.05) than those analyzed in the other PPC. This difference accentuated more after the first 60 min of exercise (Figure 1). This fact might be related with the lower dry heat exchange observed in PPC#2 and PPC#3 (Table 3) due to the thickness and air porosity of these garments (Havenith et al., 2011). The studied PPC are manufactured with highly heat-resistant fabrics (i.e., Nomex and Kevlar) mixed with fire resistant viscose, which lends PPC a greater air permeability and comfort (Yoo and Barker, 2005). The PPC#3 composition had a 10% less of viscose (Table 1), so its porosity and air permeability was reduced, which might negatively affect the dry heat exchange. The PPC#2 had a double layer in the shoulders area which increases its thickness and thermal insulation, thus limiting the dry heat exchange (Holmér, 2006). The reduced dry heat exchange and evaporative heat loss obtained in PPC#3 would explain the higher increase in gastrointestinal temperature observed in this garment (0.7°C) compared to the other ensembles (~0.3°C). No significant differences were found in evaporative heat transfer between PPC (Table 3). Probably this was related to the variability of the evaporated sweat calculation. However, the higher relative humidity observed underneath PPC#2 and PPC#3 and the lower magnitude of evaporative heat loss, suggests that the evaporative heat transfer might be decreased in both garments. Several studies have related the higher humidity in the microclimate with the lesser effective sweat evaporation (Kwon et al., 1998; Yoo and Barker, 2005; Bröde et al., 2008).

Although the lower relative humidity was analyzed in PPC#4 (Figure 2), this did not lead to increased sweat evaporation. Indeed, the lower value was found in PPC#4 (Table 2), obtaining a sweat efficiency similar to that of PPC#2 and PPC#3 (~60%). The PPC#4 composition (100% cotton) and its greater thickness might have conditioned this pattern (Yoo and Barker, 2005). It has been reported the high capacity of cotton to retain moisture and its low evaporative efficiency when sweating is abundant (Kwon et al., 1998). These results confirm previous findings (Holmér, 1985), which highlight the high capacity of synthetic fibers (e.g., polyester, nylon, aramid) to transfer moisture quickly to the outside.

A higher sweat residue in the PPC might be beneficial to reduce the subjects' thermal strain since it would increase its thermal conductivity (Chen et al., 2003; Keiser and Rossi, 2008). In addition, the body movement when walking generates air currents that improve the heat exchange by increasing the ventilation (Qian and Fan, 2009). This forced convection due to movement might have helped the sweat evaporation throughout the trials (Lotens and Havenith, 1995; Bröde et al., 2008). This would avoid an excessive increase in the subjects' thermophysiological response at the end of the trials (Figure 1). However, a high sweat residue in PPC might be a disadvantage during wildfires suppressions. In these situations wildland firefighters are exposed to both radiant and convective heat (Raimundo and Figueiredo, 2009), with heat flows that oscillate between 0.42 and 8.37 kW·m^−2^ (Mäkinen, 2005). Under this circumstance the heat transfer would be reversed, passing the body to gain heat instead of dissipating it (Holmér, 2006). Therefore, a high amount of moisture retained in the PPC might increase the risk of scalds (Keiser and Rossi, 2008). Taking into account the above, the PPC#1 would be the most advantageous garment to protect the wildland firefighters in real scenarios, since it retained ~65% less moisture content (Table 2). With this PPC subjects obtained the highest comfort (i.e., the lowest moisture sensation), which might affect the wildland firefighters' work efficiency and performance (Nayak et al., 2014).

In summary, no significant differences in the cardiorespiratory variables, blood lactate, gastrointestinal temperature, PSI and RPE between the different PPCs were found. However, our results suggest that the PPC composition affected the sweat efficiency and moisture sensation. The highest sweat efficiency and comfort were analyzed when subjects wore the PPC#1. In addition, the lowest moisture content was found in this garment. This might mean more protection for wildland firefighters in real situations, when they have to work close to the flames.

## Author contributions

Study Design: BC, JL, JV, JR. Data collection: BC, JL, JV. Data analyses: BC, JL, JV. Interpretation of the results: BC, JL, JV, JR. Manuscript writing: BC, JR. Approved the final manuscript version: BC, JL, JV, JR.

### Conflict of interest statement

The authors declare that the research was conducted in the absence of any commercial or financial relationships that could be construed as a potential conflict of interest.

## References

[B1] Baker S. J. Grice J. Roby L. Matthews C. (2000). Cardiorespiratory and thermoregulatory response of working in fire-fighter protective clothing in a temperate environment. Ergonomics 43, 1350–1358. 10.1080/001401300421798 11014757

[B2] Borg G. A. V. (1982). Psychological bases of perceived exertion. Med. Sci. Sports Exerc. 14, 48–58. 7154893

[B3] Bröde P. Havenith G. Wang X. Candas V. den Hartog E. A. Griefahn B.. (2008). Non-evaporative effects of a wet mid layer on heat transfer through protective clothing. Eur. J. Appl. Physiol. 104, 341–349. 10.1007/s00421-007-0629-y 18084775

[B4] Brotherhood J. R. Budd G. M. Hendrie A. L. Jeffrey S. E. Beasley F. A. Costin B. P. (1997). Project Aquarius 3. effects of work rate on the productivity, energy expenditure, and physiological responses of men building fireline with a rakehoe in dry eucalypt forest. Int. J. Wildl. Fire 7, 87–98. 10.1071/WF9970087

[B5] Bruce R. A. (1971). Exercise testing of patients with coronary artery disease. Ann. Clin. Res. 3, 323–332. 5156892

[B6] Bruce-Low S. S. Cotterrell D. Jones G. E. (2007). Effect of wearing personal protective clothing and self-contained breathing apparatus on heart rate, temperature and oxygen consumption during stepping exercise and live fire training exercises. Ergonomics 50, 80–98. 10.1080/00140130600980912 17178653

[B7] Budd G. M. Brotherhood J. R. Hendrie A. L. Jeffery S. E. Beasley F. A. Costin B. P. (1997). Project Aquarius 13. the thermal burden of high insulation and encapsulation in wildland firefighters clothing. Int. J. Wildl. Fire 7, 207–218. 10.1071/WF9970207

[B8] Chen Y. S. Fan J. Zhang W. (2003). Clothing thermal insulation during sweating. Text Res. J. 73, 152–157. 10.1177/004051750307300210

[B9] Cheuvront S. N. Kenefick R. W. Montain S. J. Sawka M. N. (2010). Mechanisms of aerobic performance impairment with heat stress and dehydration. J. Appl. Physiol. 109, 1989–1995. 10.1152/japplphysiol.00367.2010 20689090

[B10] Cuddy J. S. Sol J. A. Hailes W. S. Ruby B. C. (2015). Work patterns dictate energy demands and thermal strain during wildland firefighting. Wilderness Environ. Med. 26, 221–226. 10.1016/j.wem.2014.12.010 25772825

[B11] Dorman L. E. Havenith G. (2009). The effects of protective clothing on energy consumption during different activities. Eur. J. Appl. Physiol. 105, 463–470. 10.1007/s00421-008-0924-2 19011890

[B12] DuBois D. DuBois E. F. (1916). A formula to estimate the approximate surface area if height and weight be known. Arch. Intern. Med. 17, 863–871.

[B13] Gagge A. P. Gonzalez R. R. (1996). Mechanisms of heat exchange: biophysics and physiology, in Handbook of Physiology, Environmental Physiology, eds Fregly M. J. Blatteis C. M. (Bethesda, MD: American Physiological Society), 46–84.

[B14] Gonzalez-Alonso J. Teller C. Andersen S. L. Jensen F. B. Hyldig T. Nielsen B. (1999). Influence of body temperature on the development of fatigue during prolonged exercise in the heat. J. Appl. Physiol. 86, 1032–1039. 10066720 10.1152/jappl.1999.86.3.1032

[B15] Havenith G. (2002). Interaction of clothing and thermoregulation. Exog. Dermatol. 1, 221–230. 10.1159/000068802

[B16] Havenith G. denHartog E. Martin S. (2011). Heat stress in chemical protective clothing: porosity and vapour resistance. Ergonomics 54, 497–507. 10.1080/00140139.2011.558638 21547794

[B17] Havenith G. Heus R. (2004). A test battery related to ergonomics of protective clothing. Appl. Ergon. 35, 3–20. 10.1016/j.apergo.2003.11.001 14985136

[B18] Holmér I. (1985). Heat exchange and thermal insulation compared in woolen and nylon garments during wear trials. Text Res. J. 55, 511–518. 10.1177/004051758505500901

[B19] Holmér I. (2006). Protective clothing in hot environments. Ind. Health 44, 404–413. 10.2486/indhealth.44.404 16922184

[B20] Holmér I. Kuklane K. Gao C. (2006). Test of firefighter's turnout gear in hot and humid air exposure. Int. J. Occup. Saf. Ergon. 12, 297–305. 10.1080/10803548.2006.11076689 16984788

[B21] International Standardization Organization (ISO) (2003). Protective Clothing for Firefighters. Laboratory Test Methods and Performance Requirements for Wildland Firefighting Clothing. ISO 15384. Geneva: ISO.

[B22] Keiser C. Rossi R. (2008). Temperature analysis for the prediction of steam formation and transfer in multilayer thermal protective clothing at low level thermal radiation. Text Res. J. 78, 1025–1035. 10.1177/0040517508090484

[B23] Kofler P. Burtscher M. Heinrich D. Bottoni G. Caven B. Bechtold T.. (2015). Performance limitation and the role of core temperature when wearing light-weight workwear under moderate thermal conditions. J. Therm. Biol. 47, 83–90. 10.1016/j.jtherbio.2014.11.007 25526658

[B24] Kwon A. Kato M. Kawamura H. Yanai Y. Tokura H. (1998). Physiological significance of hydrophilic and hydrophobic textile materials during intermittent exercise in humans under the influence of warm ambient temperature with and without wind. Eur. J. Appl. Physiol. 78, 487–493. 10.1007/s004210050450 9840402

[B25] Lee J.-Y. Kim S. Jang Y.-J. Baek Y.-J. Park J. (2014). Component contribution of personal protective equipment to the alleviation of physiological strain in firefighters during work and recovery. Ergonomics 57, 1068–1077. 10.1080/00140139.2014.907449 24773624

[B26] Lotens W. A. Havenith G. (1995). Effects of moisture absorption in clothing on the human heat balance. Ergonomics 38, 1092–1113. 10.1080/00140139508925176 7758441

[B27] Mäkinen H. (2005). Firefighter's protective clothing, in Textiles for Protection, ed Scott R. A. (Cambridge, Woodhead), 622–647.

[B28] McLellan T. M. Pope J. I. Cain J. B. Cheung S. S. (1996). Effects of metabolic rate and ambient vapour pressure on heat strain in protective clothing. Eur. J. Appl. Physiol. Occup. Physiol. 74, 518–527. 8971493 10.1007/BF02376767

[B29] Montain S. J. Sawka M. N. Cadarette B. S. Quigley M. D. McKay J. M. (1994). Physiological tolerance to uncompensable heat stress: effects of exercise intensity, protective clothing, and climate. J. Appl. Physiol. 77, 216–222. 7961236 10.1152/jappl.1994.77.1.216

[B30] Nayak R. Houshyar S. Padhye R. (2014). Recent trends and future scope in the protection and comfort of fire-fighters' personal protective clothing. Fire Sci. Rev. 3, 4 10.1186/s40038-014-0004-0

[B31] Phillips M. Petersen A. Abbiss C. R. Netto K. Payne W. Nichols D.. (2011). Pack Hike Test finishing time for Australian firefighters: pass rates and correlates of performance. Appl. Ergon. 42, 411–418. 10.1016/j.apergo.2010.08.020 20888552

[B32] Poirier M. P. Meade R. D. McGinn R. Friesen B. J. Hardcastle S. G. Flouris A. D.. (2015). The influence of Arc-flash and fire- resistant clothing on thermoregulation during exercise in the heat. J. Occup. Environ. Hyg. 12, 654–667. 10.1080/15459624.2015.1029615 25898230

[B33] Qian X. Fan J. (2009). A quasi-physical model for predicting the thermal insulation and moisture vapour resistance of clothing. Appl. Ergon. 40, 577–590. 10.1016/j.apergo.2008.04.022 18835476

[B34] Raimundo A. M. Figueiredo A. R. (2009). Personal protective clothing and safety of firefighters near a high intensity fire front. Fire Saf. J. 44, 514–521. 10.1016/j.firesaf.2008.10.007

[B35] Rodríguez-Marroyo J. A. López-Satue J. Pernía R. Carballo B. García-López J. Foster C.. (2012). Physiological work demands of Spanish wildland firefighters during wildfire suppression. Int. Arch. Occup. Environ. Health 85, 221–228. 10.1007/s00420-011-0661-4 21656120

[B36] Ruby B. C. Shriver T. C. Zderic T. W. Sharkey B. J. Burks C. Tysk S. (2002). Total energy expenditure during arduous wildfire suppression. Med. Sci. Sports Exerc. 34, 1048–1054. 10.1097/00005768-200206000-00023 12048336

[B37] Saunders A. G. Dugas J. P. Tucker R. Lambert M. I. Noakes T. D. (2005). The effects of different air velocities on heat storage and body temperature in humans cycling in a hot, humid environment. Acta Physiol. Scand. 183, 241–255. 10.1111/j.1365-201X.2004.01400.x 15743384

[B38] Selkirk G. A. McLellan T. M. (2001). Influence of aerobic fitness and body fatness on tolerance to uncompensable heat stress. J. Appl. Physiol. 91, 2055–2063. 11641344 10.1152/jappl.2001.91.5.2055

[B39] Selkirk G. A. McLellan T. M. (2004). Physical Work Limits for toronto firefighters in warm environments. J. Occup. Environ. Hyg. 1, 199–212. 10.1080/15459620490432114 15204859

[B40] Sköldström B. (1987). Physiological responses of fire fighters to workload and thermal stress. Ergonomics 30, 1589–1597. 10.1080/00140138708966049 3443086

[B41] Smith D. L. Petruzzello S. J. (1998). Selected physiological and psychological responses to live-fire drills in different configurations of firefighting gear. Ergonomics 41, 1141–1154. 10.1080/001401398186441 9715673

[B42] Taylor N. A. S. Lewis M. C. Notley S. R. Peoples G. E. (2012). A fractionation of the physiological burden of the personal protective equipment worn by firefighters. Eur. J. Appl. Physiol. 112, 2913–2921. 10.1007/s00421-011-2267-7 22143844

[B43] Tikuisis P. McLellan T. M. Selkirk G. (2002). Perceptual vs. physiological heat strain during exercise-heat stress. Med. Sci. Sports Exerc. 34, 1454–1461. 10.1097/00005768-200209000-00009 12218738

[B44] Wang F. Gao C. Kuklane K. Holmér I. (2013). Effects of various protective clothing and thermal environments on heat strain of unacclimated men: the PHS (predicted heat strain) model revisited. Ind. Health 51, 266–274. 10.2486/indhealth.2012-0073 23385435

[B45] Wegesser T. C. Pinkerton K. E. Last J. A. (2009). California wildfires of 2008: coarse and fine particulate matter toxicity. Environ. Health Perspect. 117, 893–897. 10.1289/ehp.0800166 19590679 PMC2702402

[B46] Wen S. Petersen S. McQueen R. Batcheller J. (2015). Modelling the physiological strain and physical burden of chemical protective coveralls. Ergonomics 58, 2016–2031. 10.1080/00140139.2015.1051595 26135878

[B47] Yoo S. Barker R. L. (2005). Comfort properties of heat resistant protective workwear in varying conditions of physical activity and environment. part II: perceived comfort response to garments and its relationship to fabric properties. Text. Res. J. 75, 531–539. 10.1177/0040517505054190

